# Correlation between pedometer and the Global Physical Activity Questionnaire on physical activity measurement in office workers

**DOI:** 10.1186/1756-0500-7-280

**Published:** 2014-05-03

**Authors:** Ekalak Sitthipornvorakul, Prawit Janwantanakul, Allard J van der Beek

**Affiliations:** 1 Interdisciplinary Program of Biomedical Sciences, Faculty of Graduate School, Chulalongkorn University, Bangkok, Thailand; 2 Department of Physical Therapy, Faculty of Allied Health Sciences, Chulalongkorn University, Bangkok, Thailand; 3 Department of Public and Occupational Health, EMGO Institute for Health and Care Research, VU University Medical Center, Amsterdam, The Netherlands; 4 Body@Work, Research Center on Physical Activity, Work and Health, TNO-VUmc, Amsterdam, The Netherlands

## Abstract

**Background:**

This study aimed to examine the correlation of physical activity levels assessed by pedometer and those by the Global Physical Activity Questionnaire (GPAQ) in a population of office workers.

**Methods:**

A cross-sectional study was conducted on 320 office workers. A self-administered questionnaire was distributed to each office worker by hand. Physical activity level was objectively assessed by a pedometer for 7 consecutive days and subjectively assessed by the GPAQ. Based on the pedometer and GPAQ outcomes, participants were classified into 3 groups: inactive, moderately active, and highly active.

**Results:**

No correlation in the physical activity level assessed by the pedometer and GPAQ was found (*r*_s_ = .08, *P* = 0.15). When considering the pedometer as the criterion for comparison, 65.3% of participants had underestimated their physical activity level using the GPAQ, whereas 9.3% of participants overestimated their physical activity level.

**Conclusions:**

Physical activity level in office workers assessed by a subjective measure was greatly different from assessed by an objective tool. Consequently, research on physical activity level, especially in those with sedentary lifestyle, should consider using an objective measure to ensure that it closely reflects a person’s physical activity level.

## Background

Daily physical activity, which is activity at rather low to moderate levels, when performed sufficiently is widely known to have important health benefits [1]. Insufficient levels of daily physical activity has been linked to several chronic health problems, including diabetes mellitus [2,3], ischemic heart disease, stroke, breast cancer, colon/rectal cancer [2], and chronic musculoskeletal complaints [4]. Musculoskeletal disorders are common among the working population [5] and office workers are one of occupations that suffer from musculoskeletal symptoms with a high proportion experiencing symptoms in the spine [6]. Apart from personal suffering and impaired quality of life in general, musculoskeletal symptoms in office workers can lead to sickness absence and reduced work effectiveness [7,8].

Sedentary behaviour is defined as having an energy expenditure lower than or equal to 1.5 Metabolic Equivalent of Task (MET), while in a sitting or reclining position. Sedentary behaviour is distinct from physical inactivity; a person can be performing sedentary activities, but still be physically active according to physical activity guidelines [9]. For instance, an office worker sitting 7 hours per day at work and watching 2 hours of television per day, can still commute by bicycle and exercise for half an hour three days per week. This person performs sedentary activities on the one hand, while is physically active on the other. Associations have been found between sedentary behaviour and diabetes mellitus, cardiovascular disease, and all-cause mortality, independent of the level of physical activity [9-13]. However, the evidence for these associations is less strong for occupational sedentary behaviour in specific [14]. Thus, the notion of potential health benefits is of high interest, not only for increased physical activity but also for decreased sedentary behavior.

Common measurement methods of physical activity level include self-reported questionnaire, interviewing, and objective instrument (i.e. an accelerometer or a pedometer). Pedometer is one of objective tools for assessing physical activity [15]. Step count from a pedometer is used to represent physical activity level. However, a pedometer can neither measure non-ambulatory activity nor intensity or type of physical activity. Still, several studies have shown that a pedometer provide a valid and accurate measure of physical activity level in free-living conditions [16-18].

The global physical activity questionnaire (GPAQ) is one of commonly used questionnaires for assessing physical activity level in population-based studies due to their low cost, low participant burden and ease of administration [19-22]. The GPAQ consists of 16 questions assessing a typical week’s activity undertaken in different domains, such as work, transport, and leisure or recreation. Fifteen items assessing physical activity, while one additional item pays attention to time spent in sedentary activities. The GPAQ has been reported to be a reliable questionnaire for physical activity measurement [23,24].

Different occupations are exposed to different working conditions and the nature of the work influences the health of workers [25]. Therefore, an expectation of the same health benefits of increased physical activity for all those in differing occupations would be irrational. Thus, research on the health benefits of increased physical activity should take the impact of work status into account. The GPAQ records physical activity in the work domain, in which both vigorous and moderate activities are assessed. However, sedentary workers, such as office workers, may only have light activities at work, consequently affecting the accuracy of the GPAQ. To date, no study has investigated the correlation of physical activity level measured by a subjective and objective tool in sedentary workers. Thus, the aim of this study was to examine the correlation of physical activity level measured by the GPAQ and a pedometer among sedentary workers. Knowledge obtained from this study aims to provide researchers with guidance on the appropriate measurement method of physical activity level in those with sedentary jobs.

## Methods

### Participants and procedures

A cross-sectional study was conducted on a convenience sample of office workers recruited from workplaces in Bangkok. Office workers were defined as those working in an office environment with their main tasks involving computer use, participation in meetings, presentations, reading, and telephoning [26]. Subjects were excluded if they had had neck or low back symptoms during the previous 3 months with reporting pain intensity greater than 30 millimeters (mm) on a 100-mm visual analogue scale, reported pregnancy, or had a history of surgery, trauma, or accidents in the spinal region. Subjects who had been diagnosed with congenital anomaly of the spine, rheumatoid arthritis, infection of the spine and discs, ankylosing spondylitis, spondylolisthesis, spondylosis, tumor, systemic lupus erythymatosus, or osteoporosis were also excluded from the study.

Before principal data collection, repeatability of data from both the pedometer and GPAQ (see: measures) was assessed in 10 office workers. Each subject was tested on two occasions separated by an interim of 7 days between measurements.

Principal data collection consisted of assessments using pedometers and questionnaires. First, each participant was given a pedometer with an instruction to carry it for 7 consecutive days. Second, a self-administered questionnaire, i.e. the GPAQ, was distributed to each office worker by hand and the researcher returned to collect the completed questionnaire around 20 minutes later. The body weight and body height of all participants were obtained to calculate the body mass index. Written informed consent was obtained from all participants. The study was approved by the Chulalongkorn University Human Ethics Committee.

### Measures

#### Pedometer

The pedometer used in the present study was the Yamax Digiwalker CW-700 (Yamax, Tokyo, Japan). The Yamax pedometer is accurate and reliable for counting steps [17,27]. Each participant was asked to wear the pedometer for 7 consecutive days in order to record daily steps during these days. Participants were instructed to carry the pedometer on the right side of the belt, in the midline of the thigh, from getting up in the morning until going back to bed at night. Participants were allowed to remove the pedometer only while immersing the body in water. Participants received a short massage via mobile phone everyday to remind them to wear the pedometer during the 7-day period of physical activity measurement. Average steps per day recorded by the pedometer were calculated for each participant, who had at least four daily measurements [28-30]. Participants were classified, according to their average daily steps, as inactive (<5,000 steps per day), moderately active (5,000 to 9,999), and highly active (≥10,000) [31].

#### Questionnaire

The self-administered questionnaire consisted of two sections in order to gather data on demographics and physical activity level (the GPAQ) [20]. Participants reported duration (min) and frequency (time/week) of physical activity participation in three domains; activity at work, travel to and from places, and recreational activities. Total physical activities were calculated by the sum of the total metabolic equivalents (MET) minutes of activity computed for each domain. For the calculation of a categorical indicator, the total time spent in physical activity during a typical week, the numbers of days as well as the intensity of the physical activity are taken into account. Total physical activity scores from the GPAQ were used to divide participants into 3 groups: inactive, moderately active, and highly active. The criteria for these levels are shown below.

##### Highly active

A person reaching any of the following criteria is classified in this category: vigorous-intensity activity on at least 3 days achieving a minimum of at least 1,500 MET-minutes per week OR 7 or more days of any combination of walking, moderate- or vigorous-intensity activities achieving a minimum of at least 3,000 MET-minutes per week.

##### Moderately active

A person not meeting the criteria for the “Highly active” category, but meeting any of the following criteria is classified in this category: 3 or more days of vigorous-intensity activity of at least 20 minutes per day OR 5 or more days of moderate-intensity activity or walking of at least 30 minutes per day OR 5 or more days of any combination of walking, moderate- or vigorous- intensity activities achieving a minimum of at least 600 MET-minutes per week.

##### Inactive

A person not meeting any of the above mention criteria falls in this category.

### Statistical analyses

For the reliability study, the intraclass correlation coefficient (ICC) was calculated for the average daily steps measured by pedometer. The Kendall’s tau-b was calculated for the physical activity level measured by the GPAQ.

Descriptive statistics were calculated for all variables. Kolmogorov-Smirnov’s test was performed to check the distribution of the data. Due to the non-normal distribution of data, the Spearman’s rank correlation test was applied to assess the association between the physical activity level assessed by pedometer and the GPAQ. All statistical analyses were performed using SPSS statistical software, version 16.0 (SPSS Inc, Chicago, IL, USA). Statistical significance was set at the 5% level.

## Results

The reliability results demonstrated good reliability for the pedometer with an ICC (3,2) score of 0.77 as well as the GPAQ outcome with a Kendall’s tau-b score of 0.89.

In total, 320 office workers participated in the study. Table 1 displays the descriptive variables including the demographic data, level of physical activity from the pedometer and GPAQ. There was no correlation in the physical activity level assessed by the pedometer and GPAQ (*r*_s_ = 0.08, *P* = 0.15).

**Table 1:** Demographic and physical activity levels of participating office workers (n = 320)

**Characteristics**	**N (%)**	**Mean (SD)**
*Demographic characteristics*		
Age		34.8 (6.2)
Gender		
Male	64 (20.0)	
Female	256 (80.0)	
Body mass index (kg/m2)		23.6 (4.9)
Education		
Lower than Bachelor’s degree	47 (14.7)	
Bachelor’s degree	224 (70.0)	
Higher than Bachelor’s degree	49 (15.3)	
*Physical activity levels*		
Pedometer (steps/day)		
Inactive (<5,000)	20 (6.2)	
Moderately active (5,000-9,999)	210 (65.6)	
Highly active (≥10,000)	90 (28.1)	
GPAQ		
Inactive	193 (60.3)	
Moderately active	90 (28.1)	
Highly active	37 (11.6)	

When stratified subjects by age, the result showed significant but low correlation between the physical activity level assessed by the pedometer and GPAQ in those aged between 20–29 years (n = 77, *r*_s_ = 0.27, *P* = 0.01). No correlation was found for participants aged between 30–39 years (n = 155, *r*_s_ = -0.01, *P* = 0.87) and over 40 years (n = 88, *r*_s_ = 0.09, *P* = 0.39).

**Figure 1:**
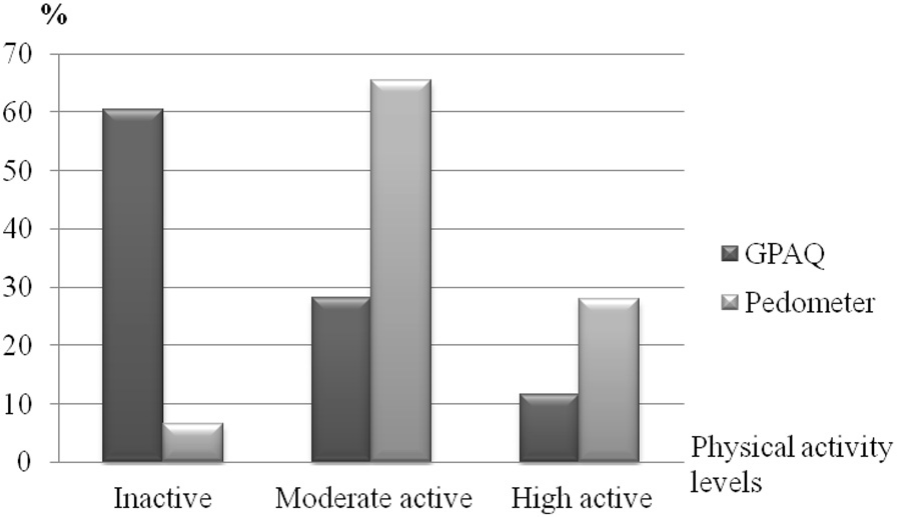
Proportion of participants according to physical activity level assessed by the GPAQ and pedometer (n = 320).

Figure 1 shows the proportion of participants for each physical activity level assessed by the pedometer and GPAQ. When considering the pedometer as the criterion for comparison, 65.3% of participants had underestimated their physical activity level using the GPAQ, whereas 9.3% of participants overestimated their physical activity level (Table 2).

**Table 2:** Distribution of participants according to physical activity level assessed by the GPAQ and pedometer (n = 320)

	**Pedometer**			**Total n (%)**
**GPAQ**	**Inactive n (%)**	**Moderately active n (%)**	**Highly active n (%)**	
Inactive	15 (4.7)^a^	129 (40.3)^b^	49 (15.3)^b^	193 (60.3)
Moderately active	3 (0.9)^c^	56 (17.5)^a^	31 (9.7)^b^	90 (28.1)
Highly active	2 (0.6)^c^	25 (7.8)^c^	10 (3.1)^a^	37 (15.2)
Total	20 (6.2)	210 (65.6)	90 (28.1)	320 (100)

*Abbreviation:* GPAQ, the Global Physical Activity Questionnaire. ^a^Pedometer as the criterion for comparison: correct estimator, ^b^Pedometer as the criterion for comparison: under-estimator, ^c^Pedometer the criterion for comparison: over-estimator.

## Discussion

We found that physical activity level assessed by a subjective tool, such as the GPAQ, did not correlate to that assessed by an objective tool, such as a pedometer, in an office worker population with sedentary lifestyle. Only a small improvement of correlation in physical activity level measured by the pedometer and GPAQ was found when stratified participants by age. Participants were likely to underestimate their physical activity level when using the GPAQ compared to a pedometer. The finding is in line with recent studies investigating the association between physical activity level measured by subjective and objective tools. Kl et al. [31] found a small association between physical activity level measures by the pedometer and GPAQ in a general population, while Cleland et al. [30] found significant but modest correlation between physical activity level measured by pedometer and the International Physical Activity Questionnaire in a general population.

On the one hand, all questions in the GPAQ, which evaluates physical activity in work, transport, and leisure or recreation domains, focus on long duration and moderate- to high-intensity activities, for example, “Does your work involve vigorous-intensity activity that causes large increases in breathing or hearth rate for at least 10 minutes continuously?” or “How many days do you do vigorous-intensity activities as part of your work?” or “How much time do you spend doing vigorous-intensity activities at work on a typical day?” On the other hand, a pedometer counts the steps during all types of activities for a whole day. The target population of the present study consisted of office workers, whose job characteristic is sedentary. Tudor-Locke and Myers [28], in their review about physical activity measurement among sedentary adults, reported that self-reported measures tend to capture structured activities of long duration and high intensity, whereas pedometers can capture incidental activities of shorter duration and lower intensity. As a result, no correlation in the physical activity level assessed by the pedometer and GPAQ was found among office workers.

Physical activity level measured by the GPAQ was underestimated when using the pedometer as a criterion for comparison. Verbunt et al. [15], in their review of assessment methods of physical activity level, indicated that self-report measurements may lead to either under- or overestimation of physical activity level. Recently, Kl et al. [31], in their validity study, found that the GPAQ had overestimated physical activity level when using pedometer as a criterion for comparison in a general population. The discrepancy between the present study and their previous study may be due to difference in studied population. In the study by Kl et al. [31], participants’ occupation was not controlled, while in the present study participants were healthy office workers. Physical activity engagement is different across occupational categories. Blue-collar workers showed significantly higher occupational physical activity and were thus involved in more moderate- and high-intensity activity types, while white-collar workers spent most of their time at work sitting and performing light occupational activities [32]. In the present study, most office workers reported no moderate or vigorous physical activity at work when completing the GPAQ. Consequently, using the GPAQ to evaluate physical activity levels in office workers would likely lead an underestimation of physical activity level when using the pedometer as a criterion for comparison. Given the recent insights regarding the adverse health effects of sedentary behaviour, future intervention studies might aim at changing occupational sedentary behaviour into light physical activities at work. Underestimation of occupational activities of short duration and low intensity would be particularly problematic in such intervention studies, since potential beneficial effects might remain unnoticed.

Small correlation between the pedometer and GPAQ was found in younger office workers. One possible explanation for small improvement in the correlation among younger participants may relate to the fact that physical activity is dependent on age or, in other words, the probability of physical inactivity increases proportionally with increasing age [33]. The magnitude of underestimation of physical activity level measured by the GPAQ compared to the pedometer may be reduced in those with moderate to high physical activity. Since this is the first study to investigate the correlation of physical activity level measured by the GPAQ and a pedometer in those with sedentary jobs, further study is required before firm conclusions can be drawn.

A number of previous studies on physical activity level employed self-reported questionnaire or interviewing [34]. The findings of the present study showed that objective methods reported different results from those obtained from subjective methods, especially in those with sedentary lifestyle. Self-report measurements may lead to incorrect physical activity level, which may result in bias in the association between physical activity and interested outcomes. An objective measure is preferable for assessing physical activity level. Its advantages include having greater validity with minimal burden on participants, although high cost and restricted registration time can still be barriers. Future research should attempt to use an objective measure to evaluate physical activity level.

### Strengths and limitations of the study

The major strength of this study is the relative homogeneity of the population. Indexes that classify pedometer-determined physical activity are proposed for those free of chronic diseases and disabilities [31]. Only healthy office workers were included in this study. However, there are three main methodological limitations that should be taken into consideration when interpreting the results of the present study. First, the pedometer is generally not sensitive to non-ambulatory activities. Despite the limitations of a pedometer, use of this device is a relatively simple way to monitor the performance-based physical activity status of healthy people. Second, body mass index was not controlled among participants in the present study. Previous studies had indicated that the pedometer could not accurately record steps for obese people [35,36]. Although hardly any obese workers were included in the present study, future study with a control of body mass index among participants is recommended to confirm the findings of this study. Lastly, no physical verification method was implemented to check if participants used the pedometer as instructed, although they were reminded through a short massage via mobile phone everyday to wear the pedometer during the 7-day period of physical activity measurement. Future studies should consider inclusion of a physical verification method of using the pedometer to increase data accuracy.

## Conclusions

To conclude, the subjective measurement of physical activity level, i.e. the GPAQ, was not associated with the objective measurement, i.e. a pedometer, in office workers. Self-reported measurement likely led to underestimation of physical activity level. Therefore, further research on physical activity, particularly in those with sedentary lifestyle, should consider using objective measures rather than those based on subjective self-reports. Additional study is necessary to validate the above conclusion.

## Competing interests

The authors declare that there are no conflicts of interest.

## Authors' contributions

The authors have contributed in the following way: ES provided concept/research design, data collection, data analysis and manuscript writing. PJ provided concept/research design, data analysis and manuscript writing. PP and AJB provided concept/research design and manuscript writing. All authors read and approved the final manuscript.
